# Alcohol Impairs Bioenergetics and Differentiation Capacity of Myoblasts from Simian Immunodeficiency Virus-Infected Female Macaques

**DOI:** 10.3390/ijms25042448

**Published:** 2024-02-19

**Authors:** Danielle E. Levitt, Brianna L. Bourgeois, Keishla M. Rodríguez-Graciani, Patricia E. Molina, Liz Simon

**Affiliations:** 1 Department of Physiology, School of Medicine, Louisiana State University Health Sciences Center, New Orleans, LA 70112, USA; danielle.levitt@ttu.edu (D.E.L.); bbou11@lsuhsc.edu (B.L.B.); krodr7@lsuhsc.edu (K.M.R.-G.); pmolin@lsuhsc.edu (P.E.M.); 2 Comprehensive Alcohol-HIV/AIDS Research Center, Louisiana State University Health Sciences Center, New Orleans, LA 70112, USA; 3 Department of Kinesiology and Sport Management, Texas Tech University, Lubbock, TX 79409, USA

**Keywords:** alcohol use disorder, glycolysis, mitochondria, ethanol, antiretroviral therapy, HIV, myopathy, skeletal muscle, satellite cell

## Abstract

Alcohol misuse and HIV independently induce myopathy. We previously showed that chronic binge alcohol (CBA) administration, with or without simian immunodeficiency virus (SIV), decreases differentiation capacity of male rhesus macaque myoblasts. We hypothesized that short-term alcohol and CBA/SIV would synergistically decrease differentiation capacity and impair bioenergetic parameters in female macaque myoblasts. Myoblasts from naïve (CBA^−^/SIV^−^), vehicle [VEH]/SIV, and CBA/SIV (N = 4–6/group) groups were proliferated (3 days) and differentiated (5 days) with 0 or 50 mM ethanol (short-term). CBA/SIV decreased differentiation and increased non-mitochondrial oxygen consumption rate (OCR) versus naïve and/or VEH/SIV. Short-term alcohol decreased differentiation; increased maximal and non-mitochondrial OCR, mitochondrial reactive oxygen species (ROS) production, and aldolase activity; and decreased glycolytic measures, ATP production, mitochondrial membrane potential (ΔΨm), and pyruvate kinase activity. Mitochondrial ROS production was closely associated with mitochondrial network volume, and differentiation indices were closely associated with key bioenergetic health and function parameters. Results indicate that short-term alcohol and CBA non-synergistically decrease myoblast differentiation capacity. Short-term alcohol impaired myoblast glycolytic function, driving the bioenergetic deficit. Results suggest potentially differing mechanisms underlying decreased differentiation capacity with short-term alcohol and CBA, highlighting the need to elucidate the impact of different alcohol use patterns on myopathy.

## 1. Introduction

Approximately one-quarter to one-third of adults in the United States report past-month alcohol misuse (i.e., binge or heavy drinking) [1,2] and an estimated 28.5 million (~15% of adults) have an alcohol use disorder (AUD) [3]. Globally, the prevalence of AUD is more than double among people with HIV (PWH) [4] compared to the general population [5]. Alcohol misuse has multisystemic adverse consequences [6,7] and may be especially harmful for PWH [8]. Myopathy, or decreased skeletal muscle (SKM) mass and function, affects nearly half of those with AUD [9] and has a high prevalence among PWH [10,11]. Myopathy, in turn, is associated with increased mortality in several clinical conditions [12], highlighting the importance of maintaining SKM mass and function.

Although SKM has emerged as a key tissue impacted by alcohol use [9,13,14,15], the underlying mechanisms of alcohol-associated myopathy and whether these differ among PWH remain to be completely elucidated. Muscle precursor cells (myoblasts) are critical for the maintenance and regeneration of muscle mass [16,17], and their bioenergetic state supports their myogenic progression [18,19,20]. Therefore, bioenergetic dyshomeostasis in SKM and myoblasts may contribute to alcohol-associated myopathy with or without HIV. Among PWH, we found that alcohol misuse was associated with poorer myoblast bioenergetic health and altered SKM mitochondrial gene expression [21]. Using a highly relevant preclinical model of HIV pathogenesis and associated comorbidities, we also have previously shown that myoblasts from chronic binge alcohol (CBA)-administered, SIV-infected, antiretroviral therapy (ART)-treated male rhesus macaques had decreased maximal mitochondrial respiration compared to those from alcohol- and SIV-naïve macaques despite myoblast expansion in the absence of alcohol [22]. At higher alcohol concentrations (100 mM), mitochondrial bioenergetic function in C2C12 myotubes was also impaired [23]. At lower concentrations (50 mM), we observed a shift in bioenergetic function away from glycolysis and toward mitochondrial metabolism in myoblasts from alcohol- and SIV-naïve macaques [24]. However, specific contributions of each bioenergetic system and potential impairments in mitochondrial and glycolytic measures that may have been impacted remain unknown. Moreover, direct comparison of different alcohol exposure paradigms, reflecting different alcohol use patterns, has not been performed.

Findings from CBA-administered animals allow for the assessment of lasting effects of chronic alcohol misuse (i.e., after a period of abstinence) since myoblasts are cultured in the absence of alcohol. On the other hand, treating myoblasts with alcohol in vitro in the short-term (i.e., alcohol in cell culture removed ≤1 h prior to assay or harvest) reflects impacts of recent alcohol use, which may or may not extend to longer-term effects. Our previous work has used only one of the alcohol administration paradigms (i.e., CBA or short-term in vitro alcohol); however, we have not directly compared the effects of both or examined them in combination. The combination allows for modeling continued alcohol use in someone with a history of misuse, and short-term use could accentuate chronic effects. Since myopathy can result from short-term and chronic alcohol misuse, examining how these different patterns of alcohol misuse may contribute to the development of myopathy is critical for appropriate early intervention [25].

Data on the impact of alcohol and HIV infection are particularly sparse for females even though people assigned female at birth comprise more than 20% of PWH [26]. Based on our previous work in SIV-infected male macaques and SIV-naïve male and female macaques, we hypothesized that short-term alcohol and CBA/SIV would decrease differentiation capacity and impair multiple bioenergetic functional parameters, and that the effects would be additive with the combination of short-term alcohol treatment (mimicking acute, recent exposure) and chronic in vivo binge alcohol administration (CBA/SIV). Myoblasts rely heavily on anaerobic glycolysis during proliferation and early differentiation [18,27], and differential reliance on distinct metabolic pathways during myogenic progression appears critical for terminal fusion [28,29,30]. Moreover, we previously found that exposing proliferating myoblasts from naïve macaques to short-term alcohol treatment shifted their bioenergetic phenotype away from glycolysis in association with decreased differentiation [24]. Therefore, we aimed to further dissect this bioenergetic impairment during proliferation as a potential contributing factor to decreased myoblast differentiation.

## 2. Results

### 2.1. Myoblast Differentiation Indices

Myoblasts were isolated from *vastus lateralis* muscle of naïve macaques (no SIV or CBA exposure) and SIV-infected macaques administered a water vehicle (VEH/SIV) or CBA (CBA/SIV) for 14.5 months. Myoblasts at P4 were proliferated for 3 days and differentiated for 5 days in 0 or 50 mM ethanol (EtOH). Myoblasts from each macaque were grown in both short-term EtOH conditions. To quantify differentiation capacity, myoblasts were methanol-fixed and stained using HEMA3 stains. We assessed myotube differentiation indices from each short-term EtOH condition and in vivo treatment group (naïve, VEH/SIV, CBA/SIV) at differentiation day 5.

**Figure 1:**
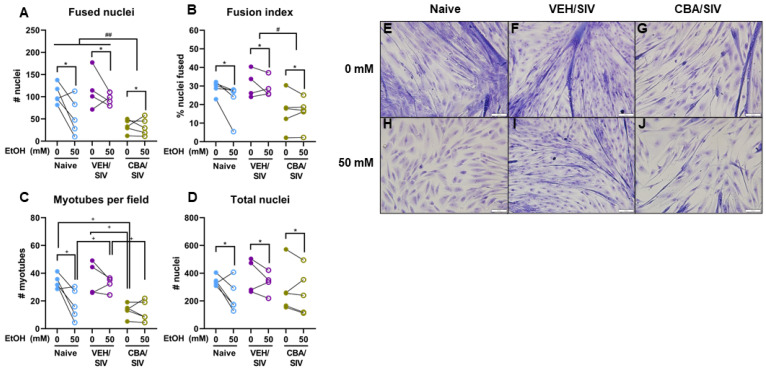
Quantification of myotube differentiation indices. Fused nuclei (**A**), fusion index (**B**), myotubes per field (**C**), and total nuclei (**D**) after 3 days of proliferation plus 5 days of differentiation with representative images (20×) of myoblasts from macaques in the naïve (N = 5), vehicle (VEH)/simian immunodeficiency virus (SIV)(N = 4), and chronic binge alcohol (CBA)/SIV (N = 5) groups cultured with 0 mM (**E**–**G**) or 50 mM (**H**–**J**) EtOH. Scale bars (lower right in each image) indicate 50 µm. Main effects of short-term EtOH, * *p* < 0.05; main effect of group (naïve, VEH/SIV, CBA/SIV) with post-hoc pairwise comparisons, # *p* < 0.05, ## *p* < 0.01; short-term EtOH × group (naïve, VEH/SIV, CBA/SIV) interaction effect with post-hoc pairwise comparisons, + *p* < 0.05. Lengths of bracket legs indicate direction of differences.

For fused nuclei (Figure 1A), there was a main effect of short-term EtOH (*p* = 0.02, η^2^ = 0.40, large). Across all groups, short-term 50 mM EtOH treatment decreased fused nuclei (d = 0.8, large) compared to 0 mM EtOH. There was also a main effect of group (*p* < 0.01, η^2^ = 0.68, large). Regardless of short-term EtOH, there were fewer fused nuclei in the CBA/SIV vs. naïve group (*p* < 0.01, d = 2.1, large) and in the CBA/SIV vs. VEH/SIV group (*p* < 0.001, d = 3.1, large). No EtOH × group interaction effect was observed for fused nuclei.

For the fusion index (Figure 1B), there was a main effect of short-term EtOH (*p* = 0.05, η^2^ = 0.30, large). Across all groups, short-term 50 mM EtOH treatment decreased the fusion index (d = 0.4, medium) compared to 0 mM EtOH. There was also a main effect of group (*p* = 0.04, η^2^ = 0.44, large). Regardless of short-term EtOH, the fusion index was decreased in the CBA/SIV vs VEH/SIV group (*p* = 0.02, d = 1.9, large). No EtOH × group interaction effect was observed for the fusion index. 

For myotubes per field, there was a significant short-term EtOH x group interaction effect (*p* = 0.05, η^2^ = 0.42, large; Figure 1C). For cells treated with 0 mM EtOH, there were fewer myotubes per field in the CBA/SIV vs naïve group (*p* < 0.01, d = 4.1, large) and the CBA/SIV vs. VEH/SIV group (*p* < 0.001, d = 2.5, large). For cells treated with 50 mM EtOH, there were fewer myotubes per field in the naïve vs VEH/SIV group (*p* = 0.03, d = 1.7, large) and in the CBA/SIV vs. VEH/SIV group (*p* < 0.01, d = 3.0, large). Compared with 0 mM, 50 mM EtOH significantly decreased myotubes per field in the naïve group (*p* < 0.01, d = 1.9, large) but not in the VEH/SIV or CBA/SIV groups. 

For total nuclei (Figure 1D), there was a main effect of short-term EtOH (*p* = 0.03, η^2^ = 0.36, large). Regardless of group, short-term 50 mM EtOH treatment decreased total nuclei (d = 0.5, medium) compared to 0 mM EtOH. No main effect of group or EtOH × group interaction effect was observed for total nuclei. Representative images of myotubes from each EtOH condition and group are shown in Figure 1E–J.

### 2.2. Mitochondrial Stress Test

**Figure 2:**
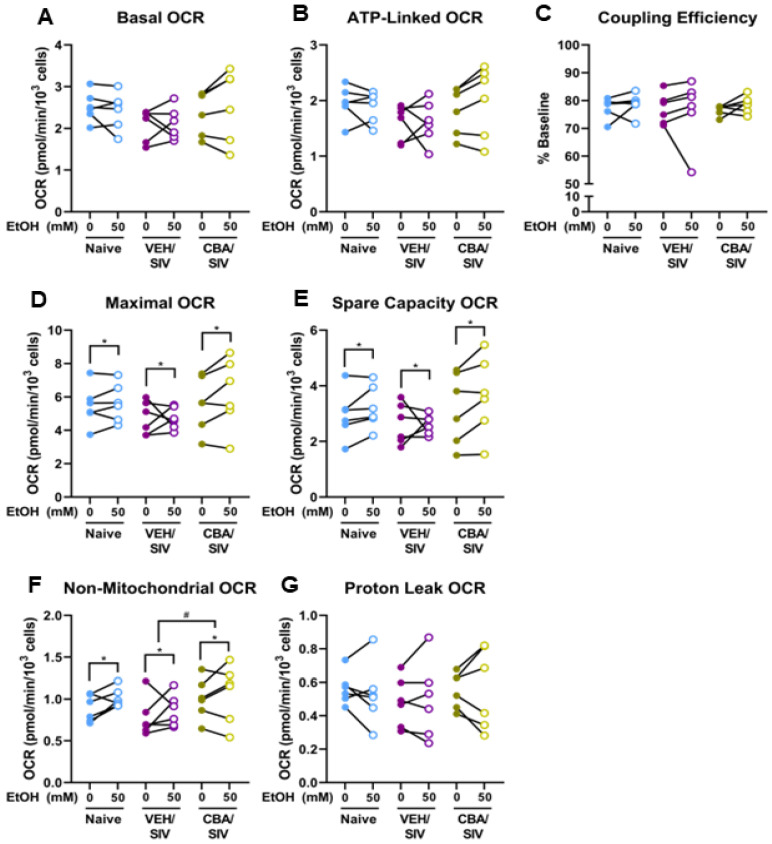
Mito Stress Test parameters. Basal (**A**) and ATP-linked (**B**) oxygen consumption rate (OCR) and coupling efficiency (**C**); ATP-linked OCR/baseline OCR × 100), maximal OCR (**D**) and spare capacity (**E**), non-mitochondrial OCR (**F**), and proton leak-linked OCR (**G**). Myoblasts from macaques in the naïve (N = 6), vehicle (VEH)/simian immunodeficiency virus (SIV) (N = 6), and chronic binge alcohol (CBA)/SIV (N = 6) groups were proliferated for 3 days with 0 mM or 50 mM EtOH. Main effects of short-term EtOH, * *p* < 0.05; main effect of group (naïve, VEH/SIV, CBA/SIV) with post-hoc pairwise comparisons, # *p* < 0.05. Lengths of bracket legs indicate direction of differences.

We performed a Mito Stress Test to examine mitochondrial function under resting and stressed conditions using myoblasts isolated from each group (naïve, VEH/SIV, and CBA/SIV) treated with 0 or 50 mM EtOH for 3 days. We measured basal oxygen consumption rate (OCR; Figure 2A), ATP-linked OCR (quantified after ATP synthase inhibition with oligomycin) (Figure 2B), and coupling efficiency (calculated as the percentage of basal OCR attributable to ATP production) (Figure 2C). No main effect of short-term EtOH, group (naïve, VEH/SIV, CBA/SIV), or short-term EtOH × group interaction effect was observed for these measures. After the addition of carbonyl cyanide-p-trifluoromethoxyphenylhydrazone (FCCP) to uncouple the inner mitochondrial membrane, main effects of short-term EtOH were observed for maximal oxygen consumption (*p* = 0.05, η^2^ = 0.31, large, Figure 2D) and spare capacity (*p* = 0.02, η^2^ = 0.43, large, Figure 2E). Regardless of group, short-term 50 mM EtOH treatment increased these measures (maximal oxygen consumption: d = 0.4, small; spare capacity: d = 0.5, medium) compared to 0 mM EtOH. After adding rotenone and antimycin A to inhibit electron transport chain complexes I and III to quantify negative indicators of bioenergetic health (non-mitochondrial OCR and proton leak-linked OCR), main effects of short-term EtOH (*p* = 0.01, η^2^ = 0.44, large) and group (*p* = 0.04, η^2^ = 0.45, large) were observed for non-mitochondrial OCR (Figure 2F). Regardless of group, short-term 50 mM EtOH treatment increased non-mitochondrial OCR (d = 0.7, medium) compared to 0 mM EtOH. Regardless of short-term EtOH, non-mitochondrial OCR was greater in the CBA/SIV vs VEH/SIV group (*p* = 0.01, d = 2.0, large). No differences were observed for proton leak (Figure 2G). A schematic diagram of an OCR trace for the Mito Stress Test and the resulting parameters reported herein is available in Figure S1 (Supplementary Materials).

**Figure 3:**
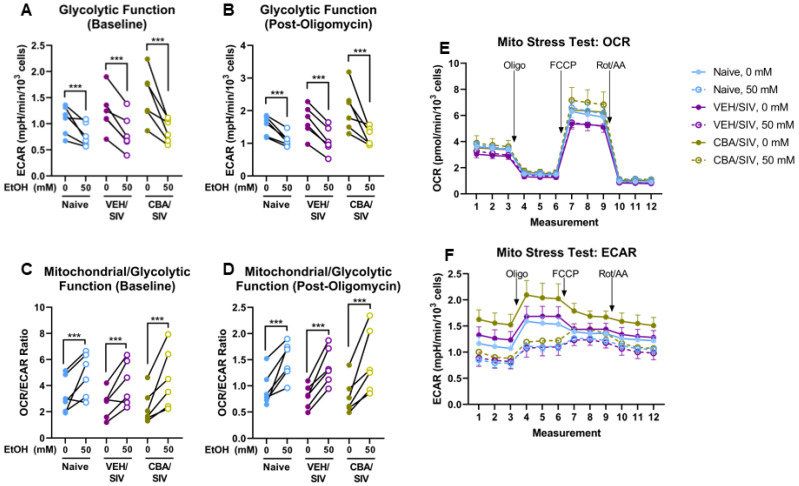
Bioenergetic phenotype indices. Glycolytic function measured as extracellular acidification rate (ECAR) at baseline (**A**) and after oligomycin administration (**B**), the relative ratio or mitochondrial to glycolytic function quantified as the ratio of oxygen consumption (OCR) to extracellular acidification (ECAR) rates at baseline (**C**) and after oligomycin administration (**D**), and OCR (**E**) and ECAR (**F**) throughout the assay with indications for injections of oligomycin (Oligo), carbonyl cyanide-p-trifluoromethoxyphenylhydrazone (FCCP), and rotenone/antimycin A (Rot/AA). Myoblasts from macaques in the naïve (N = 6), vehicle (VEH)/simian immunodeficiency virus (SIV) (N = 6), and chronic binge alcohol (CBA)/SIV (N = 6) groups were proliferated for 3 days with 0 mM or 50 mM EtOH. Main effects of short-term EtOH, *** *p* < 0.001. Lengths of bracket legs indicate direction of differences.

To determine the overall bioenergetic phenotype, extracellular acidification rate (ECAR; indicative of glycolytic function) and the OCR/ECAR ratio (indicative of mitochondrial vs glycolytic function) at baseline and after ATP synthase inhibition were used. Main effects of short-term EtOH were observed for ECAR at baseline (*p* < 0.001, η^2^ = 0.85, large, Figure 3A) and after ATP synthase inhibition (*p* < 0.001, η^2^ = 0.87, large, Figure 3B), and for the ratio of OCR to ECAR at baseline (*p* < 0.001, η^2^ = 0.76, large, Figure 3C) and after ATP synthase inhibition (*p* < 0.001, η^2^ = 0.81, large, Figure 3D). Regardless of group, short-term 50 mM EtOH treatment decreased each of these measures (baseline ECAR: d = 1.2, large; ECAR after ATP synthase inhibition: d = 1.3, large; baseline OCR/ECAR: d = 1.2, large; OCR/ECAR after ATP synthase inhibition: d = 1.5, large) compared to 0 mM EtOH. No main effect of group (naïve, VEH/SIV, CBA/SIV) or EtOH × group interaction effect was observed for bioenergetic phenotype. The OCR and ECAR traces throughout the assay are shown by group in Figure 3E,F. 

### 2.3. Glycolysis Stress Test

**Figure 4:**
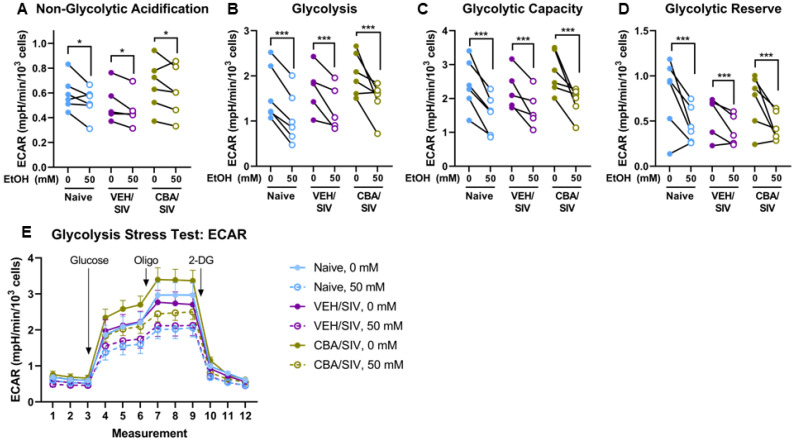
Glycolysis stress test parameters. Non-glycolytic acidification (**A**), basal glycolysis (**B**), glycolytic capacity (**C**), and glycolytic reserve (**D**) measured as extracellular acidification rates (ECAR) and ECAR traces throughout the assay (**E**) with indications for injections of glucose, oligomycin (Oligo), and 2-deoxyglucose (2-DG). Myoblasts from macaques in the naïve (N = 6), vehicle (VEH)/simian immunodeficiency virus (SIV) (N = 5), and chronic binge alcohol (CBA)/SIV (N = 6) groups were proliferated for 3 days with 0 mM or 50 mM EtOH. Main effects of short-term EtOH, * *p* < 0.05; *** *p* < 0.001. Lengths of bracket legs indicate direction of differences.

A glycolysis stress test was used to identify specific changes in glycolytic measures using myoblasts isolated from each group (naïve, VEH/SIV, and CBA/SIV) treated with 0 or 50 mM EtOH for 3 days. Main effects of short-term EtOH were observed for non-glycolytic acidification (*p* = 0.02, η^2^ = 0.42, large, Figure 4A), baseline glycolysis (*p* < 0.001, η^2^ = 0.76, large, Figure 4B), glycolytic capacity (*p* < 0.001, η^2^ = 0.80, large, Figure 4C), and glycolytic reserve (*p* < 0.001, η^2^ = 0.66, large, Figure 4D). Regardless of group (naïve, VEH/SIV, CBA/SIV), short-term 50 mM EtOH treatment decreased non-glycolytic acidification (d = 0.4, small), baseline glycolysis (d = 1.1, large), glycolytic capacity (d = 1.4, large), and glycolytic reserve (d = 1.3, large) compared to 0 mM EtOH. No main effects of group or EtOH × group interaction were observed for glycolysis stress test outcomes. The ECAR traces throughout the assay are shown by group in Figure 4E. A schematic diagram of an ECAR trace for the Glycolysis Stress Test and the resulting parameters reported herein are available in Figure S2 (Supplementary Materials).

### 2.4. ATP Production Rates

**Figure 5:**
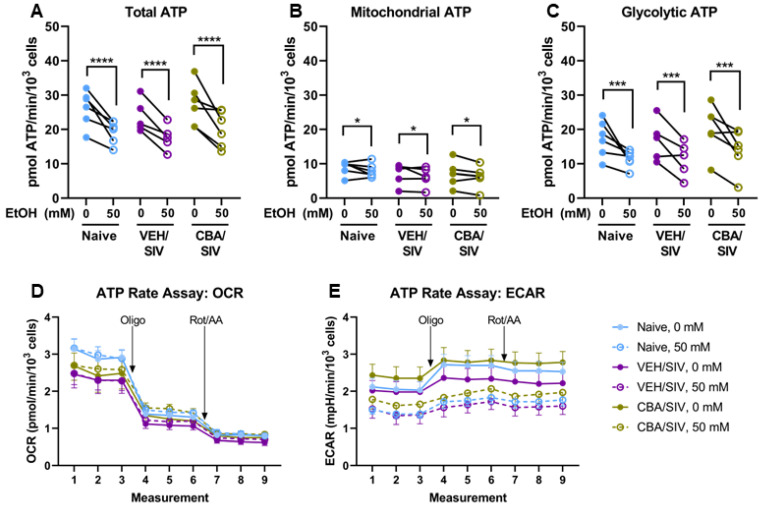
ATP rate assay parameters. Total ATP (**A**), ATP derived from mitochondrial metabolism (**B**) and anaerobic glycolysis (**C**), and oxygen consumption ((**D**); OCR) and extracellular acidification ((**E**); ECAR) rates throughout the assay, with indications for injections of oligomycin (Oligo) and rotenone/antimycin A (Rot/AA). Myoblasts from macaques in the naïve (N = 6), vehicle (VEH)/simian immunodeficiency virus (SIV) (N = 5), and chronic binge alcohol (CBA)/SIV (N = 6) groups were proliferated for 3 days with 0 mM or 50 mM EtOH. Main effects of short-term EtOH; * *p* < 0.05; *** *p* < 0.001; **** *p* < 0.0001. Lengths of bracket legs indicate direction of differences.

To dissect differences in ATP production attributable to mitochondrial and glycolytic pathways, an ATP rate assay was performed using myoblasts isolated from each group (naïve, VEH/SIV, and CBA/SIV) treated with 0 or 50 mM EtOH for 3 days. Main effects of short-term EtOH were observed for total ATP production rate (*p* < 0.001, η^2^ = 0.76, large, Figure 5A), ATP produced via mitochondrial (*p* = 0.04, η^2^ = 0.34, large, Figure 5B) and glycolytic (*p* < 0.001, η^2^ = 0.65, large, Figure 5C) metabolism. Short-term 50 mM EtOH treatment decreased the total ATP production rate (d = 1.3, large), glycolysis-derived ATP (d = 1.0, large), and mitochondrial-derived ATP (d = 0.5, medium) compared to 0 mM EtOH. No main effect of group (naïve, VEH/SIV, CBA/SIV) or short-term EtOH × group interaction was observed for ATP production rate outcomes. The OCR and ECAR traces throughout the assay are shown by group in Figure 5D,E.

### 2.5. Mitochondrial Health

**Figure 6:**
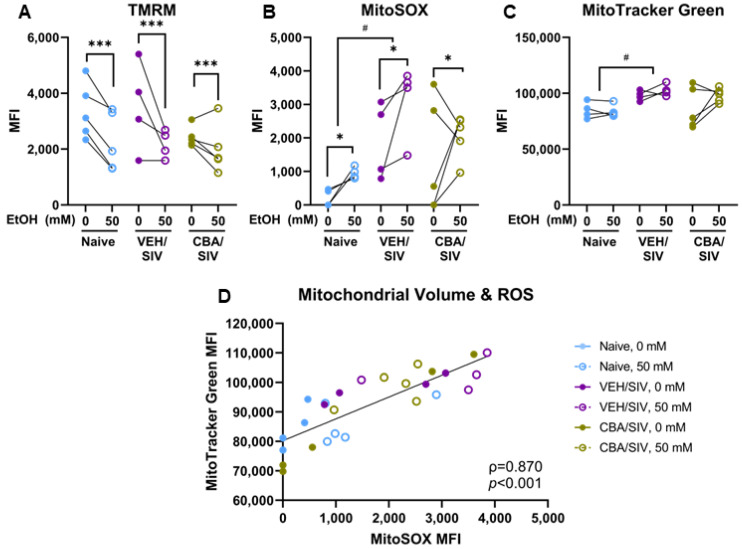
Mitochondrial health parameters. Mean fluorescence intensity (MFI) of tetramethylrhodamine methyl ester (TMRM) for mitochondrial membrane potential (ΔΨm; (**A**)), MitoSOX for mitochondrial reactive oxygen species (ROS; (**B**)), and MitoTracker Green for mitochondrial network volume (**C**) measured using flow cytometry. Mitochondrial network volume was significantly associated with ROS (**D**). Myoblasts from macaques in the naïve (N = 5 [**A**] or 4 [**B**–**D**]), vehicle (VEH)/simian immunodeficiency virus (SIV) (N = 4), and chronic binge alcohol (CBA)/SIV (N = 5) groups were proliferated for 3 days with 0 mM or 50 mM EtOH. Main effects of EtOH, * *p* < 0.05, *** *p* < 0.001, main effect of group with post-hoc pairwise comparisons, # *p* < 0.05. Lengths of bracket legs indicate direction of differences.

We used flow cytometric analyses to evaluate mitochondrial membrane potential (ΔΨm), reactive oxygen species (ROS) production, and network volume to assess the health of the myoblast mitochondrial network in myoblasts isolated from each group (naïve, VEH/SIV, and CBA/SIV) treated with 0 or 50 mM EtOH. MitoSOX mean fluorescence intensity (MFI) was below the detectable limit in four samples; these values were set to zero. Main effects of short-term EtOH were observed for ΔΨm (*p* < 0.001, η^2^ = 0.66, large, Figure 6A) and ROS (*p* = 0.034, η^2^ = 0.63, large, Figure 6B) but not mitochondrial network volume (Figure 6C). Regardless of group (naïve, VEH/SIV, CBA/SIV), short-term 50 mM EtOH treatment decreased ΔΨm (d = 1.0, large) and increased ROS production (d = 0.7, medium). Main effects of group were observed for mitochondrial ROS (*p* = 0.02, η^2^ = 0.54, large, Figure 6B) and network volume (*p* < 0.05, η^2^ = 0.63, large, Figure 6C) but not ΔΨm. Regardless of short-term EtOH treatment, mitochondrial ROS production (d = 4.8, large) and network volume (d = 4.8, large) were greater in the VEH/SIV group compared to the naïve group. No short-term EtOH × group interaction effects were observed for these mitochondrial health measures. Across all samples, there was a significant, positive association between mitochondrial network volume and ROS (*p* < 0.001, ρ = 0.87, large, Figure 6D).

### 2.6. Glycolytic Enzyme Activity

**Figure 7:**
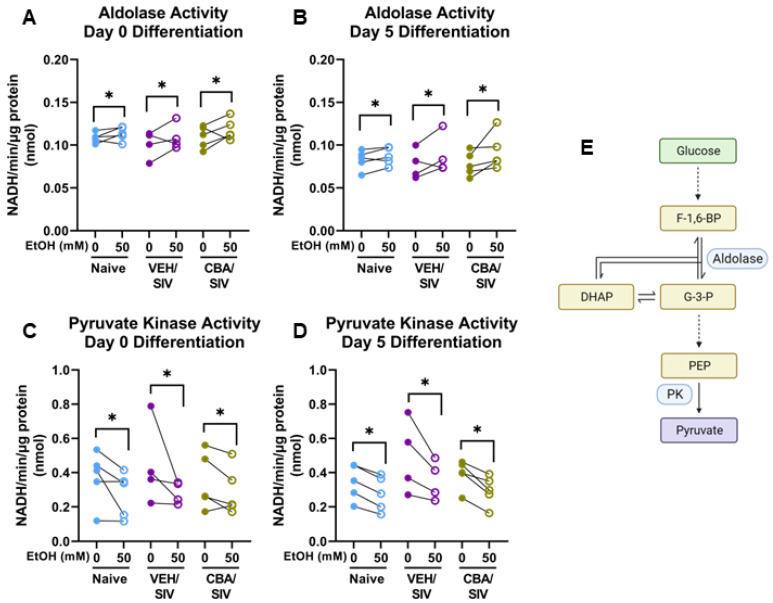
Activity of select glycolytic enzymes. Aldolase (**A**,**B**) and pyruvate kinase (**C**,**D**) activity in myoblasts from macaques in the naïve (N = 5), vehicle (VEH)/simian immunodeficiency virus (SIV) (N = 4), and chronic binge alcohol (CBA)/SIV (N = 5) groups after 3 days of proliferation (day 0 differentiation, (**A**,**C**)) and 5 days of differentiation (**B**,**D**) with 0 mM or 50 mM EtOH. Simplified schematic of the first 10 steps of anaerobic glycolysis (**E**) showing steps catalyzed by aldolase and pyruvate kinase (PK). Fructose-1,6-bisphosphate (F-1,6-BP); dihydroxyacetone phosphate (DHAP); glyceraldehyde-3-phosphate (G3P); phosphoenolpyruvate (PEP); dashed arrows indicate multiple steps between glycolysis intermediates. Main effects of short-term EtOH, * *p* < 0.05. Lengths of bracket legs indicate direction of differences.

To identify whether alterations in glycolytic enzyme activity could have had a role in the substantial decrease in glycolytic function observed, we measured the activity of two glycolytic enzymes in myoblasts isolated from each group (naïve, VEH/SIV, and CBA/SIV) treated with 0 or 50 mM EtOH for 3 days of proliferation (differentiation day 0) and 5 days of differentiation. Main effects of short-term EtOH were observed for aldolase activity at differentiation day 0 (*p* = 0.04, η^2^ = 0.34, large, Figure 7A) and day 5 (*p* = 0.01, η^2^ = 0.46, large, Figure 7B). Regardless of group (naïve, VEH/SIV, CBA/SIV), short-term 50 mM EtOH treatment increased aldolase activity at both time points (d = 0.6, medium, for both) vs 0 mM EtOH. Main effects of short-term EtOH were also observed for pyruvate kinase activity at differentiation day 0 (*p* = 0.01, η^2^ = 0.45, large, Figure 7C) and day 5 (*p* < 0.001, η^2^ = 0.78, large, Figure 7D). Regardless of group, short-term 50 mM EtOH treatment decreased pyruvate kinase activity at both time points (day 0: d = 0.7, medium; day 5: d = 0.8, large) compared to 0 mM EtOH. No main effect of group (naïve, VEH/SIV, CBA/SIV) or EtOH × group interaction was observed for glycolytic enzyme activity. A diagram showing the glycolytic steps catalyzed by aldolase and pyruvate kinase is shown in Figure 7E.

### 2.7. Correlations between Differentiation Indices and Bioenergetics Parameters

**Figure 8:**
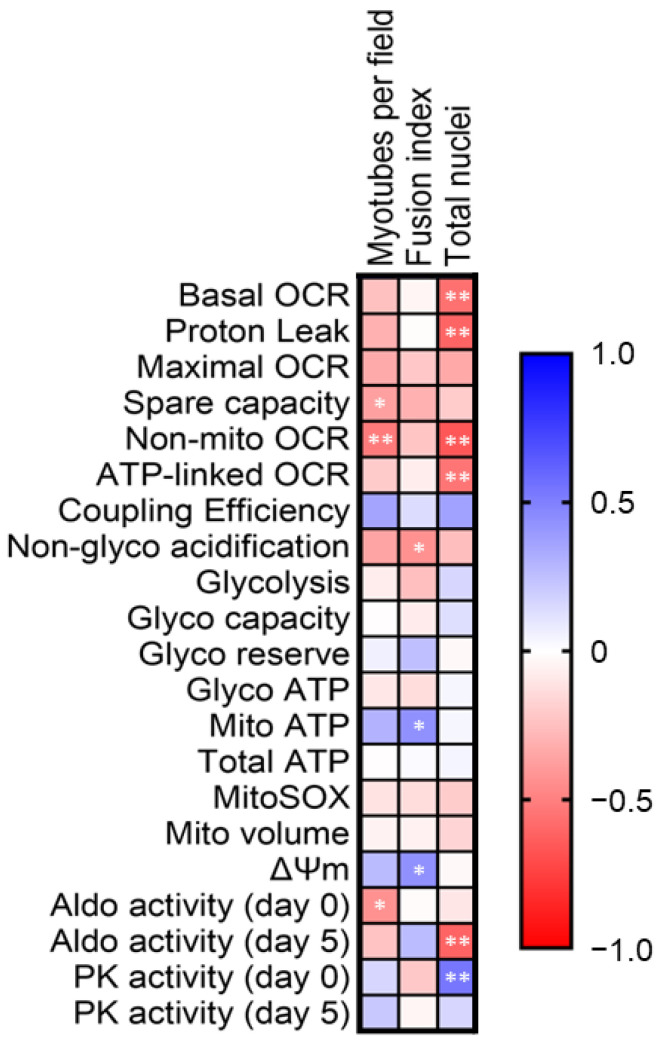
Associations between differentiation indices and bioenergetics parameters. Spearman correlations were run between raw values for key differentiation indices and bioenergetics parameters for samples collected from all groups and conditions. Oxygen consumption rate (OCR); pyruvate kinase (PK). * *p* < 0.05, ** *p* < 0.01.

To uncover potential explanatory mechanisms for the observed effects, we examined the relationships between key differentiation indices and key bioenergetic parameters by Spearman correlations (Figure 8). All samples were analyzed together to determine factors that may have contributed to impaired differentiation capacity regardless of EtOH exposure paradigm. Significant correlations were observed between myotubes per field and spare capacity (*p* = 0.05, ρ = −0.38, medium), non-mitochondrial OCR (*p* < 0.01, ρ = −0.53, large), and aldolase activity at differentiation day 0 (*p* = 0.02, ρ = −0.44, medium); between fusion index and non-glycolytic acidification (*p* = 0.02, ρ = −0.44, medium), mitochondrial ATP production (*p* = 0.02, ρ = 0.44, medium), and ΔΨm (*p* = 0.04, ρ = 0.43, medium); and between total nuclei and basal OCR (*p* < 0.01, ρ = −0.56, large), proton leak (*p* < 0.001, ρ = −0.60, large), non-mitochondrial OCR (*p* < 0.001, ρ = −0.66, large), ATP-linked OCR (*p* < 0.01, ρ = −0.55, large), aldolase activity at differentiation day 5 (*p* < 0.001, ρ = −0.61, large), and pyruvate kinase activity at differentiation day 0 (*p* < 0.01, ρ = 0.54, large). 

## 3. Discussion

Herein, we report for the first time that CBA (reflecting the lasting effects of alcohol) and short-term in vitro alcohol (reflecting current or recent alcohol use) non-synergistically decrease differentiation capacity of myoblasts from SIV-infected female macaques. However, only short-term in vitro alcohol decreased myoblast glycolytic function, mitochondrial membrane potential, and mitochondrial ROS. This differential response suggests potentially divergent mechanisms of alcohol-mediated decreased differentiation capacity. These data further our understanding of decreased myogenic differentiation with alcohol misuse and provide evidence to support critical examination of the underlying mechanisms with different alcohol use paradigms. Understanding such mechanisms would help improve alcohol-mediated myopathy, characterized by SKM weakness, injury, and atrophy, since myoblast differentiation and fusion supports muscle repair after injury [31] and rebuilding after atrophy [32]. 

### 3.1. Differentiation Indices

Short-term alcohol and CBA/SIV independently decreased measures of myoblast fusion into myotubes. The finding that myoblast fusion index was lower in CBA/SIV versus VEH/SIV groups suggests that chronic alcohol and not SIV and ART negatively impacts fusion. These findings are consistent with our previous reports of decreased fusion index with short-term alcohol in myoblasts from alcohol- and SIV-naïve male and female macaques [24] and CBA in SIV-naïve male macaques [33]. Total nuclei were decreased with short-term alcohol. This decrease could have been due to increased cell death, decreased proliferation since proliferating myoblasts rely heavily on anaerobic glycolysis [28,34], or a combination of these factors. Dissecting this finding is the subject of ongoing experiments. However, there was no decrease in total nuclei with CBA/SIV alone, and the magnitude of the decrease with short-term EtOH was not sufficient to fully explain the decreased fusion observed since the fusion index corrects for total nuclei. 

Although not measured in vivo after short-term EtOH, the findings are consistent with reports of decreased expression of myogenic genes in whole SKM after chronic in vivo EtOH [35]. However, the results contrast with our findings from SIV-infected males, where myotube density, an additional differentiation index, was decreased in VEH and CBA-administered macaques compared to SIV-naïve controls [36]. Sex differences in decreased SKM mass with HIV serostatus have previously been reported [37], where males but not females had lower SKM mass versus seronegative controls after adjusting for lifestyle factors, including alcohol use. It is possible that exposure to female sex hormones, including estrogen, could provide sufficient protection against HIV-related muscle loss, especially with less myotoxic ART drugs such as emtricitabine or tenofovir [38], whereas alcohol could be a more potent stimulus for decreased differentiation capacity and muscle mass regardless of sex. 

### 3.2. Bioenergetic Health and Function

Bioenergetic function supports myogenic progression, where myoblasts rely heavily on anaerobic glycolysis during proliferation and early differentiation [18]. The results of the present study show that short-term alcohol decreased all measures of glycolysis in proliferating myoblasts regardless of CBA and SIV. Moreover, the total rate of basal ATP production was decreased with short-term alcohol. Although a small yet significant decrease in mitochondrial ATP production was observed, there was a much more substantial decrease in the rate of glycolytic ATP production, suggesting that the bioenergetic deficit observed with short-term alcohol was primarily driven by decreased glycolytic function. Glycolytic capacity and reserve were also decreased with short-term alcohol, indicating a decreased ability for myoblasts to increase glycolytic function with increased demand for anaerobic energy production [28,34,39]. 

Although there was a decrease in basal mitochondrial ATP production rate with short-term alcohol, maximal mitochondrial respiration increased. These findings are aligned with previous results from our laboratory [24] and suggest a mitohormetic effect with short-term alcohol at a concentration of 50 mM, a physiologically relevant dose [40]. This could allow myotubes to rely more heavily on mitochondrial metabolism during times of increased energetic demands, which is particularly important given impaired glycolytic capacity and reserve. Given glycolytic deficits together with apparent mitohormesis and little evidence of decreased mitochondrial bioenergetic function despite indicators of impaired mitochondrial network health, there may be differences in fuel substrate preference with short-term EtOH. This will be the subject of future investigations. Mitohormesis with acute alcohol could be due to an altered redox state [41] requiring an adaptive increase in NAD^+^ levels [42,43,44] and mitochondrial content [21,45], as observed in various cell types. This is in contrast to decreased maximal respiration in myotubes treated with 100 mM EtOH [23], which likely induces greater oxidative stress (e.g., an approximately 3-fold increase in mitochondrial superoxide versus less than double with 50 mM EtOH in the present study) such that the mitochondria can no longer adapt. Maximal respiration was also decreased in myoblasts from CBA/SIV male macaques versus naïve male controls [22], but similar effects were not observed in female macaques in the present study. This suggests a possible sex difference in myotube mitochondrial resilience to CBA and SIV. These findings add an additional layer of complexity to the concept of sex dimorphism in SKM mitochondria [46,47,48]. 

Although myoblast bioenergetic functional changes were primarily due to short-term alcohol in the present study, increased non-mitochondrial oxygen consumption was observed following short-term alcohol and in myoblasts from CBA/SIV animals, suggesting immediate and lasting poorer bioenergetic health with alcohol [49]. This finding is supported by increased non-mitochondrial oxygen consumption in myoblasts from PWH with alcohol misuse [21]. Increased ROS production and decreased ΔΨm with short-term alcohol in the present study likely contributed to the observed increased non-mitochondrial oxygen consumption, findings supported by prior work [23]. ROS production was closely associated with increased mitochondrial volume, suggesting a larger, less healthy mitochondrial network. Although we did not observe increased mitochondrial ROS production with chronic alcohol and SIV in the present study, the increased non-mitochondrial OCR could have originated from ROS outside the mitochondria. Additionally, MitoSOX specifically detects mitochondrial superoxide, and in the CBA/SIV condition, superoxide may have been converted to hydrogen peroxide since increased mitochondrial superoxide dismutase has been observed with chronic alcohol and with 100 mM EtOH in vitro [23,45,50]. A sustained increase in whole-cell ROS with chronic alcohol may have mitigated any myoblast mitohormetic adaptations observed with short-term alcohol. 

### 3.3. Glycolytic Enzyme Activity

Given the substantial decreases in myoblast glycolytic parameters, the activity of glycolytic enzymes previously reported to be decreased with alcohol feeding in rodent SKM were assessed [51]. Contrary to findings in chronic alcohol-fed rats, aldolase activity was increased with short-term alcohol. As shown in the schematic provided in Figure 7E, aldolase catalyzes the cleavage of fructose 1,6-bisphosphate into dihydroxyacetone phosphate (DHAP) and glyceraldehyde 3-phosphate (G-3-P). In the canonical glycolytic pathway, DHAP is converted to an additional G-3-P molecule, and both are finally converted to pyruvate via pyruvate kinase. Alternatively, DHAP [52] and G-3-P [53] serve as precursors for triacylglycerol synthesis. Given the decreased pyruvate kinase activity with short-term alcohol that was consistent with decreased glycolysis, the increased aldolase activity observed could potentially be related to increased triacylglycerol synthesis. This hypothesis is supported by population-based studies showing greater circulating triacylglycerol among people with high levels of alcohol intake [54,55]. Such effects of alcohol on triacylglycerol synthesis in myoblasts will be the subject of future investigations. Pyruvate kinase serves as a rate-limiting enzyme, and its knockdown (M2 isoform) substantially decreases glycolytic function in H1299 and HepG2 cells [56]. As pyruvate is required for conversion to lactate, it is possible that decreased pyruvate kinase activity contributed to decreased glycolytic function, capacity, and reserve. An additional fate of pyruvate is entry into the mitochondria for oxidation. It is possible that decreased pyruvate availability from the lower pyruvate kinase activity could have contributed to the decreased mitochondrial ATP production rate; however, alternative fuel sources available for use could have limited the magnitude of this decrease.

### 3.4. Associations between Differentiation Indices and Bioenergetics Parameters

Basal and ATP-linked oxygen consumption (indicating mitochondrial function), proton leak and non-mitochondrial oxygen consumption (indicating cell stress), and aldolase activity were negatively associated with total nuclei, whereas pyruvate kinase activity was positively associated with total nuclei. Total nuclei depend, in part, on myoblast proliferation. Therefore, it appears that regardless of condition, lower cellular stress and more pyruvate kinase activity are likely to support myoblast proliferation. Although the relationships were weaker, we noted inverse relationships between differentiation and spare capacity, non-mitochondrial oxygen consumption, non-glycolytic acidification, and aldolase activity at differentiation day 0. Conversely, mitochondrial ATP production and ΔΨm were positively associated with differentiation. Although no substantial relationships between glycolytic parameters and differentiation were observed, samples from all groups were analyzed together and CBA/SIV independently decreased differentiation without a concomitant decrease in glycolysis. However, there was a non-significant pattern that we speculate may reflect adaptation in the bioenergetic phenotype toward glycolysis with chronic alcohol. This may have obscured relationships between glycolytic function and decreased differentiation capacity with short-term alcohol, and we propose that there may be divergent mechanisms underlying decreased myoblast differentiation capacity with short-term and chronic alcohol. Whereas short-term alcohol impairs glycolytic function, which is critical for proliferation and early differentiation [24,39], CBA-mediated decreased differentiation could be regulated by other pathways such as decreased miR-206 and an increase in one of its targets, histone deacetylase 4 [33,36,57]. 

### 3.5. Limitations

Although the experiments performed in the present study provide critical evidence, several limitations should be considered. We did not have a CBA group without SIV and ART. Therefore, although SIV and ART alone did not appear to decrease SKM differentiation capacity in females, it is possible that chronic alcohol could impact differentiation capacity differently in SIV and ART-naïve SKM. Although determining bioenergetics differences during differentiation is warranted, the purpose of the present study was to determine whether bioenergetic changes during the proliferative phase (i.e., changes preceding differentiation) could contribute to the impaired differentiation capacity. Pyruvate kinase activity was decreased at both 0 and 5 days of differentiation, suggesting that these bioenergetic changes likely persist throughout differentiation. Additionally, we did not measure whole-cell ROS production, mitochondrial biogenesis, or cell death. However, we did not previously observe differences in mitochondrial DNA quantity with short-term alcohol treatment [24] or CBA/SIV [22]. Finally, the majority of bioenergetic effects were observed with short-term alcohol performed using ex vivo studies rather than lasting effects of CBA/SIV after cell expansion in the absence of alcohol, although differentiation decreased similarly with both in vivo and in vitro treatments. Whether the same metabolic changes would be observed in the skeletal muscle precursor cell niche in vivo after short-term alcohol remains to be measured.

## 4. Materials and Methods

**Figure 9:**
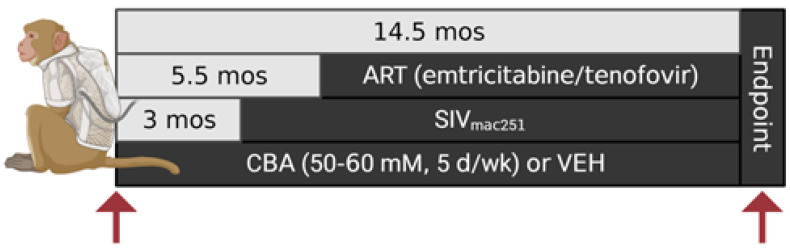
Non-human primate study design. Arrows indicate timing of muscle sampling. The first arrow indicates vastus lateralis muscle biopsy for the naïve group before initiation of CBA or VEH. The second arrow indicates muscle collection at the study endpoint for the CBA/SIV and VEH/SIV groups. Chronic binge alcohol (CBA); vehicle (isovolumetric water; VEH); simian immunodeficiency virus (SIV); antiretroviral therapy (daily subcutaneous injections; ART).

Muscle samples used to isolate myoblasts were derived from a previously described parent longitudinal study [58]. All animal experiments were approved by the Institutional Animal Care and Use Committee at Louisiana State University Health Sciences Center (LSUHSC) in New Orleans, Louisiana, and adhered to the National Institutes of Health guidelines for the care and use of experimental animals, and the methods and results are described in accordance with the ARRIVE guidelines 2.0. Adult (6–9 years old) female rhesus macaques (Macaca mulatta) were maintained on a standard chow diet before undergoing a baseline (i.e., alcohol- and SIV-naïve) vastus lateralis muscle biopsy, followed by randomization to receive chronic binge alcohol (CBA) or isovolumetric water vehicle (VEH) 5 days per week. In the CBA group, macaques were administered ethanol (30% *w*/*v*) in water over a 30 min intragastric infusion for a peak blood alcohol concentration (BAC) of 50–60 mM (approximately 0.23 g/dL) at 2 h after the start of the infusion. Three months after the initiation of CBA/VEH, macaques were intravaginally infected with SIV_mac251_. Upon reaching viral set-point 2.5 months after SIV infection, macaques were initiated on an effective daily antiretroviral therapy (ART) regimen [59] consisting of emtricitabine (FTC, 30 mg/kg) and tenofovir (20 mg/kg) subcutaneously. ART medications were provided by Gilead Sciences (Foster City, CA, USA). In the parent study, half of the macaques received ovariectomy surgery one month after ART initiation; samples from macaques post-ovariectomy were not included in the present study. Nine months after ART initiation, animals were euthanized after an overnight fast at the study endpoint according to the guidelines set forth by the American Veterinary Medical Association. SKM samples (vastus lateralis) collected at baseline and at the study endpoint were used for myoblast isolation. The total duration of CBA or VEH administration was 14.5 months (see Figure 9 for study design). Myoblasts will be referred to as “naïve” if they were derived from muscle samples collected before CBA/VEH initiation and “VEH/SIV” or “CBA/SIV” if they were derived from the study endpoint from macaques who received VEH or CBA, respectively. Myoblasts were isolated from ~50 mg of vastus lateralis tissue of 6 female macaques per group (18 total), and cells from 4–6 macaques/group were used in each experiment in the present study. Samples were labeled with IDs and group assignments were masked for experiments. 

### 4.1. Myoblast Isolation and Expansion

Primary myoblasts were isolated according to previously published methods [60]. Briefly, excess fat and blood was quickly removed from fresh muscle samples (~50 mg), and samples were minced in trypsin-EDTA (0.25%; Gibco, ThermoFisher Scientific, Waltham, MA, USA) 1:4 in Ham’s F-12 (Cytiva, Fisher Scientific, Pittsburgh, PA, USA) and agitated twice for 45 min each in 10 mL total (5 mL per agitation) of the trypsin-EDTA/Ham’s F12 mixture at room temperature. After adding 2 mL of fetal bovine serum (FBS, MilliporeSigma, Burlington, MA, USA), the mixture was centrifuged (500× *g*, 5 min, room temperature), supernatant was separated, and the cell tissue mixture was resuspended in growth medium containing 10% FBS in Ham’s F-12 with 2.5 ng/mL fibroblast growth factor (FGF, 233-FB, R&D systems, Bio-Techne, Minneapolis, MN, USA), 1× penicillin streptomycin (1% of final volume; Gibco, ThermoFisher Scientific, Waltham, MA, USA), and 4 mM L-glutamine (Gibco, ThermoFisher Scientific, Waltham, MA, USA). The cells were incubated overnight (37 °C, 5% CO_2_) before moving to a new culture dish to remove fibroblasts. The growth medium was changed every other day. The cells were grown to approximately 40–50% confluence for passage (P)0 with dense (~100% confluent) individual colonies and 80–90% confluence for subsequent passages. Experiments were performed with myoblasts at P4. 

### 4.2. Short-Term In Vitro Ethanol Treatment and HEMA3 Staining 

Myoblasts at P4 from each group (naïve, VEH/SIV, CBA/SIV) were seeded at 70,000 cells/well in 6-well plates and cultured (37 °C, 5% CO_2_) in growth media (2 mL/well) containing 0 or 50 mM ethanol (EtOH; 2.92 µL of 200-proof EtOH per 1.00 mL of media) for 3 days. Then, growth medium was replaced with differentiation medium (2% horse serum in Ham’s F-12 with 4 mM L-glutamine) containing 0 or 50 mM ethanol (EtOH) for 5 days for fusion into myotubes. This EtOH concentration is physiologically relevant [61,62,63,64] and equivalent to the peak blood EtOH level achieved daily in our in vivo model. Myoblasts from each macaque were grown in control media (0 mM EtOH) and media containing 50 mM EtOH. In both phases, EtOH-exposed cells (50 mM) were grown in an incubator containing 75 mM EtOH in the water bath to maintain the media EtOH concentration at 50 mM. This design required the experimenter to be aware of EtOH condition only during short-term EtOH treatments; however, the procedures were planned in detail a priori to minimize risk of bias. At the end of the 5-day differentiation period, myotubes were fixed in ice-cold methanol for 10 min at room temperature and stained with HEMA 3 stains according to previously published methods [60]. Briefly, 1.5 mL of HEMA 3 Solution I (Fisher Scientific, Pittsburgh, PA, USA) were added to cover cells in each well and gently agitated for 10 min at room temperature. After two washes in 1.5 mL distilled water, 1.5 mL of HEMA 3 Solution II (Fisher Scientific, Pittsburgh, PA, USA) were added to cover cells and gently agitated for 10 min at room temperature. After two further washes in 1.5 mL distilled water, the wells were dried and stored at room temperature for imaging.

### 4.3. Differentiation Indices

HEMA 3-stained cells (naïve, N = 5; VEH/SIV, N = 4; CBA/SIV, N = 5) were imaged at 20× magnification (7–8 images per well) and differentiation indices were determined as previously described [24,60]. For each image, total nuclei, total number of nuclei fused into myotubes (2+ nuclei in one cell), and number of myotubes were counted using ImageJ (https://imagej.net/ij/, accessed on 14 January 2024, National Institutes of Health, Bethesda, MD, USA) by trained investigators not involved with the study to minimize risk of bias. From these counts, myotubes per field, fused nuclei per field, and fusion index (fused nuclei/total nuclei × 100) were calculated as differentiation indices. Total nuclei were also retained as an index of proliferation. 

### 4.4. Mitochondrial Stress Test

Mitochondrial function was assessed using a Mito Stress Test (Agilent Technologies, Santa Clara, CA, USA) [24]. Myoblasts at P4 (naïve, VEH/SIV, CBA/SIV; N = 6/group) were proliferated for 2 days with 0 or 50 mM EtOH under standard cell culture conditions (5% CO_2_, 37 °C). They were then transferred to a collagen-coated 96-well Seahorse plate and seeded in triplicate at a density of 30,000 cells/well with 0 or 50 mM EtOH and maintained under standard cell culture conditions overnight. The growth media was then replaced with EtOH-free RPMI (pH 7.4 without sodium bicarbonate or phenol red; Agilent Technologies, Santa Clara, CA, USA) supplemented with sodium pyruvate (1 mM; Agilent Technologies), L-glutamine (2 mM; Agilent Technologies) and glucose (10 mM; Agilent Technologies, Santa Clara, CA, USA). The cells were incubated at 37 °C without CO_2_ for 1 h before measuring the myoblast respiratory parameters with an XFe96 Extracellular Flux Analyzer (Agilent Technologies, Santa Clara, CA, USA) according to the manufacturer’s instructions. The oxygen consumption rate (OCR) and extracellular acidification rate (ECAR) were simultaneously measured at baseline and after the sequential addition of oligomycin (1.5 µM; Agilent Technologies, Santa Clara, CA, USA), carbonyl cyanide-p-trifluoromethoxyphenylhydrazone (FCCP; 2 μM; Agilent Technologies, Santa Clara, CA, USA), and rotenone/antimycin A (0.5 μM; Agilent Technologies, Santa Clara, CA, USA). The nuclei were stained with Hoechst dye (4 µM; ThermoFisher Scientific, Waltham, MA, USA) and cell counts were captured by visualization on a BioTek Cytation 1 cell imaging multi-mode reader (BioTek, Winooski, VT, USA). Data were normalized to the cell count. Figure S1 (Supplementary Materials) shows an example OCR trace and the graphical portion associated with each parameter.

### 4.5. Glycolysis Stress Test

Glycolytic function was assessed using a Glycolysis Stress Test (Agilent Technologies, Santa Clara, CA, USA) [65]. Myoblasts at P4 (naïve, VEH/SIV, CBA/SIV; N = 6/group) were grown for 2 days and seeded in triplicate on collagen-coated 96-well Seahorse plates with 0 or 50 mM EtOH overnight as described above. The growth media was then replaced with EtOH-free, glucose-free, pyruvate-free RPMI (pH 7.4 without sodium bicarbonate or phenol red, Agilent Technologies, Santa Clara, CA, USA) supplemented with L-glutamine (2 mM). The cells were incubated at 37 °C without CO_2_ for 1 h before measuring the glycolytic parameters with an XFe96 Extracellular Flux Analyzer (Agilent Technologies, Santa Clara, CA, USA) according to the manufacturer’s instructions. The extracellular acidification rate (ECAR) was measured at baseline and after the sequential addition of glucose (10 mM; Agilent Technologies, Santa Clara, CA, USA), oligomycin (1.5 µM; Agilent Technologies, Santa Clara, CA, USA), and 2-deoxyglucose (2-DG; 50 mM; Agilent Technologies, Santa Clara, CA, USA). The nuclei were stained with Hoechst dye (ThermoFisher Scientific, Waltham, MA, USA) and the cells were counted as described above. Data were normalized to the cell count. Figure S2 (Supplementary Materials) shows an example ECAR trace and the graphical portion associated with each parameter.

### 4.6. ATP Production Rates

Total ATP production rates and the contribution of mitochondrial and glycolytic pathways were assessed using an ATP Rate Assay (Agilent Technologies, Santa Clara, CA, USA) [66]. Myoblasts at P4 (naïve, N = 6; VEH/SIV, N = 5; CBA/SIV, N = 6) were grown for 2 days and seeded in triplicate on collagen-coated 96-well Seahorse plates with 0 or 50 mM EtOH overnight as described above. The growth media was then replaced with EtOH-free RPMI (pH 7.4 without sodium bicarbonate or phenol red; Agilent Technologies, Santa Clara, CA, USA) supplemented with sodium pyruvate (1 mM; Agilent Technologies, Santa Clara, CA, USA), L-glutamine (2 mM; Agilent Technologies), and glucose (10 mM; Agilent Technologies, Santa Clara, CA, USA). The cells were incubated at 37 °C without CO_2_ for 1 h before measuring the glycolytic parameters with an XFe96 Extracellular Flux Analyzer (Agilent Technologies, Santa Clara, CA, USA) according to the manufacturer’s instructions. The oxygen consumption rate (OCR) and ECAR were simultaneously measured, and the proton efflux rate was calculated at baseline and after the sequential addition of oligomycin (1.5 μM, Agilent Technologies, Santa Clara, CA, USA) and rotenone/antimycin A (0.5 μM, Agilent Technologies, Santa Clara, CA, USA). The nuclei were stained with Hoechst dye (ThermoFisher Scientific, Waltham, MA, USA) and the cells were counted as described above. Data were normalized to the cell count.

### 4.7. Mitochondrial Health 

Commercially available probes and flow cytometry were used to assess mitochondrial membrane potential (ΔΨm) and mitochondrial reactive oxygen species (ROS) as important indicators of mitochondrial health, and to assess mitochondrial network volume to better understand adaptation during cell stress [67,68]. Myoblasts at P4 from each group were proliferated for 3 days with 0 or 50 mM EtOH and evaluated for mitochondrial membrane potential (ΔΨm) by incubating cells (naïve, N = 5; VEH/SIV, N = 4; CBA/SIV, N = 5) with tetramethylrhodamine methyl ester (TMRM, 0.1 µM, T668, ThermoFisher Scientific, Waltham, MA, USA) for 30 min at 37 °C. Separately, cells (naïve, N = 4; VEH/SIV, N = 4; CBA/SIV, N = 5) were analyzed simultaneously for mitochondrial reactive oxygen species (ROS) generation using MitoSOX Red (0.5 µM, M36008, ThermoFisher Scientific Waltham, MA, USA) and mitochondrial network volume using MitoTracker Green (0.2 µM, M7514, ThermoFisher Scientific Waltham, MA, USA) for 1 h at 37 °C. Live and dead cells were distinguished using eFluor780 (Invitrogen, Waltham, MA, USA) for all flow cytometry experiments and only results from live cells were considered for analyses. Fifty thousand events per sample were acquired on an 8-color flow cytometer (FACSCANTOII, Becton Dickinson, Franklin Lakes, NJ, USA) and the results were processed using FACSDIVA software (ver. 8.0.1 Becton Dickinson, Franklin Lakes, NJ, USA). Flow cytometry gates were defined based on fluorescence minus one (FMO) controls and are shown in Figure S3 (Supplementary Materials).

### 4.8. Glycolytic Enzyme Activity 

Commercially available assays were used to determine the activity of aldolase (MAK223, MilliporeSigma, Burlington, MA, USA) [69] and pyruvate kinase (MAK072, MilliporeSigma, Burlington, MA, USA) [70] in primary macaque myoblasts and myotubes in the naïve (N = 5), VEH/SIV (N = 4) and CBA/SIV (N = 5) groups treated in vitro with 0 or 50 mM EtOH. These enzymes were selected because of previously observed effects of chronic alcohol on their activity in SKM [51,71]. Cells were harvested for analysis after 3 days of proliferation (differentiation day 0) and differentiation day 5. The cells were lysed in the appropriate assay buffer for each kit and protein was quantified using the Pierce BCA Protein Assay Kit (ThermoFisher Scientific, Waltham, MA, USA). Samples were assayed in duplicate with 10 µg (aldolase) or 1 µg (pyruvate kinase) of protein per well. Enzyme activity was calculated according to the manufacturer’s guidelines. Values are expressed as activity per µg of protein.

### 4.9. Statistical Analyses

Data were checked for the assumption of normality using the Shapiro–Wilk test and Q-Q plots. Where normality was not met, data were log_10_-transformed to correct for this violation [72]. These transformed variables were coupling efficiency, maximal OCR, spare capacity, non-mitochondrial OCR, proton leak, ECAR at baseline and after oligomycin, and the baseline OCR/ECAR ratio. For differentiation indices and bioenergetic health and function measures, data were analyzed using a 2 (short-term EtOH) × 3 (group) analysis of variance with repeated measures on EtOH because cells from each animal were exposed to both EtOH concentrations (0 and 50 mM) for each experiment. Group (naïve, VEH/SIV, or CBA/SIV) was a between-subjects factor because all samples were derived from different macaques. For glycolytic enzyme activity, a 2 (short-term EtOH) × 3 (group) analysis of variance with repeated measures on EtOH was used to analyze data at each time point (differentiation day 0 and day 5). Because the “group” factor had 3 levels, Tukey’s HSD was used to detect the location of any main group effects. To minimize risk of bias, levels of each factor were numerically coded during analysis. Fisher’s LSD post hoc pairwise analysis was used to detect the location of any short-term EtOH × group interaction effects. Eta squared (η^2^) effect sizes were calculated for any main or interaction effects and Cohen’s d effect sizes were calculated for any pairwise differences. Data are presented as individual data points with a line connecting results from myoblasts isolated from a single individual in the control treatment (0 mM EtOH) and exposed to short-term EtOH (50 mM). Then, Spearman correlations were used to assess relationships between mitochondrial ROS and network volume and between differentiation indices and key bioenergetics parameters. For correlations, Spearman’s rho (ρ) was used as the measure of effect size. All effect size interpretations are provided in accordance with Cohen’s guidelines [73]. The alpha level of significance was set to *p* ≤ 0.05. Statistical analyses were performed using SPSS Statistics (version 29, IBM Corporation, Armonk, NY, USA).

## 5. Conclusions

The results of the present study reveal for the first time that short-term and chronic alcohol non-synergistically decrease the differentiation capacity of myoblasts from female macaques. Moreover, bioenergetic function was impaired with short-term alcohol, with a deficit in ATP production driven largely by decreased glycolytic function. Short-term in vitro alcohol treatment increased maximal mitochondrial oxygen consumption and decreased glycolytic capacity and reserve. Finally, short-term alcohol decreased pyruvate kinase activity, mitochondrial membrane potential, and mitochondrial ROS production and increased aldolase activity, suggesting possible alternative glucose fates with alcohol misuse. Although further mechanistic data are needed for confirmation, these data suggest possible divergent mechanisms underlying differentiation capacity with short-term and chronic alcohol. These findings support future investigations to examine potential therapeutic or lifestyle interventions to mitigate the adverse effects of alcohol misuse on bioenergetic function and muscle differentiation capacity, and identify targets that effectively improve functional SKM mass and quality of life in people with AUD.

## Data Availability

The data presented in this study are available on reasonable request from the corresponding author.

## Supplementary Materials

The following supporting information can be downloaded at: https://www.mdpi.com/article/10.3390/ijms25042448/s1.
