# Supplementary figures and images for: Alcohol Impairs Bioenergetics and Differentiation Capacity of Myoblasts from Simian Immunodeficiency Virus-Infected Female Macaques
Source: Int J Mol Sci. 2024 Feb 19;25(4):2448. doi: 10.3390/ijms25042448 (PMC10888832; doi:10.3390/ijms25042448)

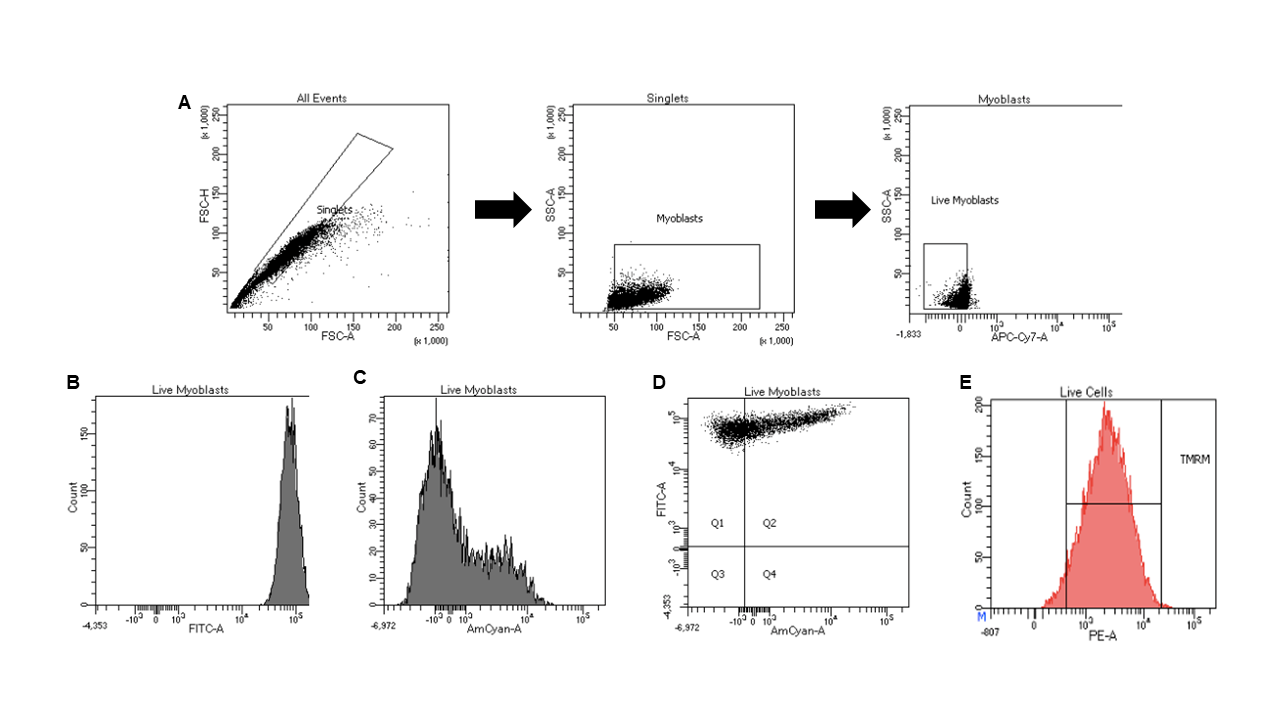

Supplement: Supplementary file 1 [file ijms-25-02448-s001.zip › Supplemental Figure 1.TIF]

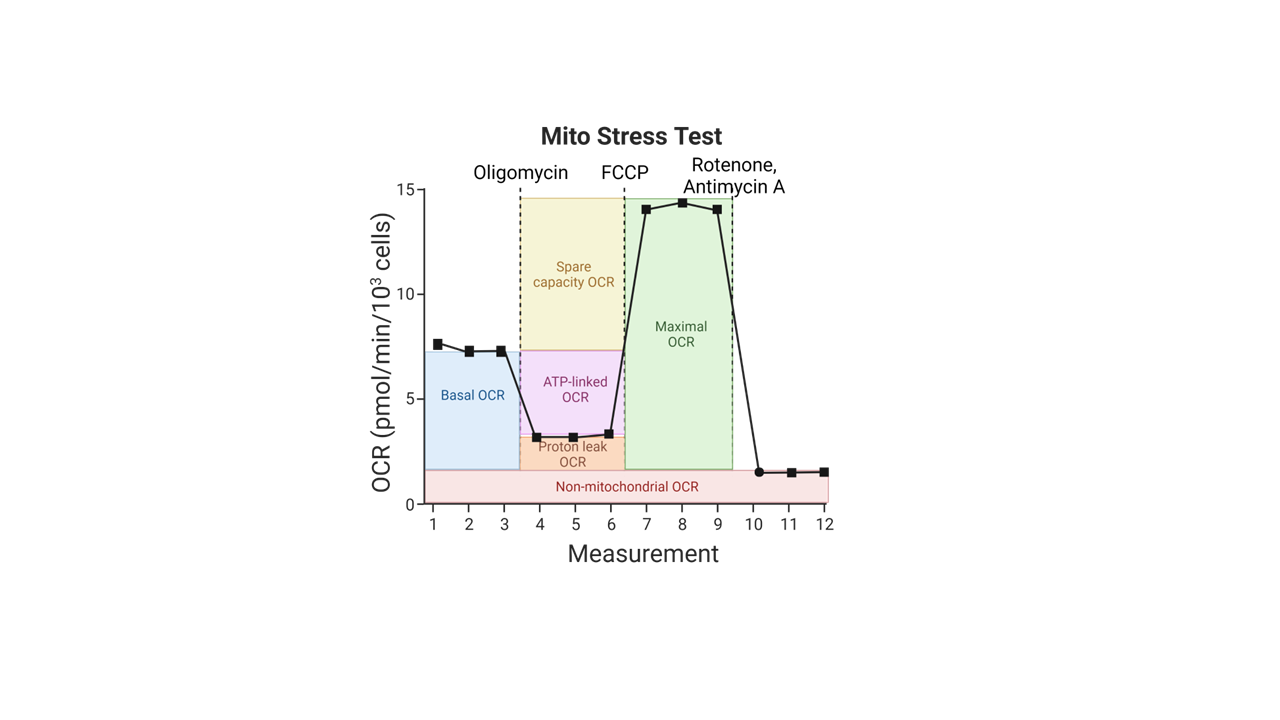

Supplement: Supplementary file 1 [file ijms-25-02448-s001.zip › Supplemental Figure 2.TIF]

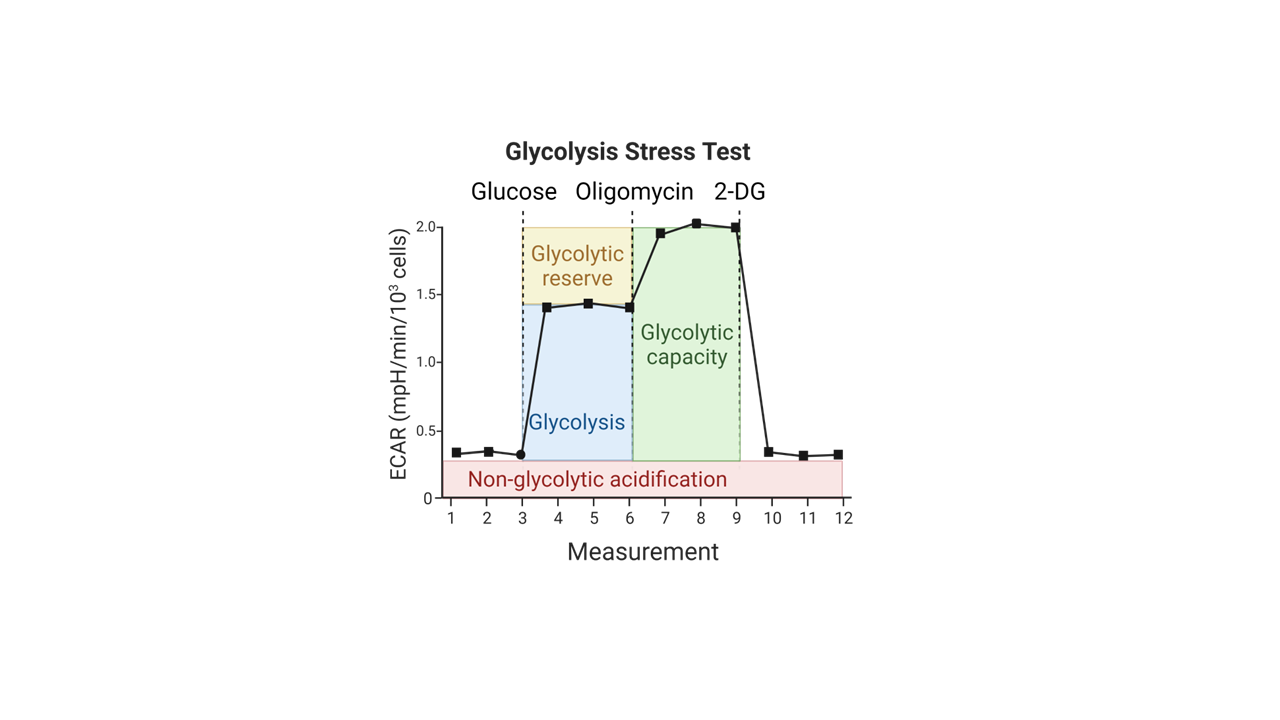

Supplement: Supplementary file 1 [file ijms-25-02448-s001.zip › Supplemental Figure 3.TIF]
